# The Haemodialysis Session Effect on the Choroidal Thickness and Retinal and Choroidal Microcirculation—A Literature Review

**DOI:** 10.3390/jcm12247729

**Published:** 2023-12-16

**Authors:** Joanna Roskal-Wałek, Joanna Gołębiewska, Jerzy Mackiewicz, Paweł Wałek, Agnieszka Bociek, Michał Biskup, Dominik Odrobina, Andrzej Jaroszyński

**Affiliations:** 1 Ophthalmology Clinic, Voivodeship Regional Hospital, 25-736 Kielce, Poland; michal.biskup@onet.pl; 2 Collegium Medicum, Jan Kochanowski University, 25-317 Kielce, Poland; pawel.walek@o2.pl (P.W.); abociek28@gmail.com (A.B.); magdale_5@hotmail.com (D.O.); andrzej.jaroszynski@ujk.edu.pl (A.J.); 3 Department of Ophthalmology, Military Institute of Aviation Medicine, 01-755 Warsaw, Poland; joanna.golebiewska@wp.pl; 4 Medical Faculty, Lazarski University, 02-662 Warsaw, Poland; 5 Department of Vitreoretinal Surgery, Medical University of Lublin, 20-079 Lublin, Poland; jerzy.mackiewicz@umlub.pl; 6 1st Clinic of Cardiology and Electrotherapy, Swietokrzyskie Cardiology Centre, 25-736 Kielce, Poland; 7 Ophthalmology Clinic Boni Fratres Lodziensis, 93-357 Łódź, Poland

**Keywords:** choroidal thickness, haemodialysis, optical coherence tomography

## Abstract

Haemodialysis (HD) is currently the most commonly used method of renal replacement therapy. The process of dialysis involves numerous changes that affect many systems, including the eye. The changes occurring in the course of HD may affect the ocular parameters, such as intraocular pressure, central corneal thickness, retinal thickness, retinal nerve fibre layer thickness, and choroidal thickness (CT). The choroid, being one of the most vascularized tissues, is characterized by the highest ratio of blood flow to tissue volume in the entire body, may be particularly susceptible to changes occurring during HD, and at the same time reflect the microcirculatory status and its response to HD. Patients with end-stage renal disease subjected to dialysis are highly susceptible to systemic microvascular dysfunction. Moreover, it is considered that the process of HD itself contributes to vascular dysfunction. Nowadays, thanks to the development of imaging techniques, the widely available optical coherence tomography (OCT) tests allow for the assessment of CT, while OCT-angiography allows for a quick, non-invasive, and repeatable assessment of the condition of retinal and choroidal microcirculation, which significantly expands our knowledge regarding the reaction of ocular microcirculation due to HD. The assessment of both retinal and choroidal circulation is even more attractive because retinal circulation is autoregulated, while choroidal circulation is mainly controlled by extrinsic autonomic innervation. Thus, assessment of the choroidal response to an HD session may provide the possibility to indirectly evaluate the functions of the autonomic system in patients subjected to HD. At a time when the importance of microcirculation in systemic and renal diseases is becoming increasingly evident, the assessment of ocular microcirculation appears to be a potential biomarker for assessing the condition of systemic microcirculation. In this work, we present a review of the literature on the effect of the HD session on CT and the retinal and choroidal microcirculation.

## 1. Introduction

Chronic kidney disease (CKD) affects approximately 13% of the world’s population, and its incidence is increasing, as is the frequency of haemodialysis (HD), which is the most frequently used method of renal replacement therapy [1,2]. The eyes and the kidneys have many structural, developmental, and functional similarities, which indicates that an ophthalmological assessment may be particularly valuable for understanding the complex haemodynamic and neurohormonal mechanisms occurring as a result of HD in patients with CKD [3].

The main purpose of HD is to control the composition and volume of systemic fluids by removing water and uraemic substances. The changes occurring in the systemic haemodynamic parameters induced by HD are relatively well known. HD usually decreases blood pressure (BP), which is associated with body weight loss and plasma volume decrease. As water and solutes are removed during HD, the serum osmolarity decreases, and plasma protein concentration increases, which leads to higher plasma colloid osmotic pressure. This increase in plasma colloid osmotic pressure leads to the occurrence of a colloid osmotic pressure gradient between the plasma and interstitial fluid, which causes water transfer from the interstitial fluid to the plasma [4].

The changes occurring in the course of HD may also affect the ocular parameters, such as intraocular pressure (IOP), central corneal thickness, anterior chamber depth, lens thickness, retinal thickness (RT), retinal nerve fibre layer (RNFL) thickness, and choroidal thickness (CT) [4,5,6,7,8,9,10,11,12,13,14,15,16,17,18,19,20,21,22,23,24].

Due to the anatomical structure and innervation of the choroid, the effect of HD is especially interesting. The choroid, being one of the most vascularized tissues, is characterized by the highest ratio of blood flow to tissue volume in the entire body, may be particularly susceptible to changes occurring during HD, and at the same time reflect the microcirculatory status and its response to HD [16,25]. Moreover, choroidal circulation is mainly controlled by extrinsic autonomic innervation, where the activation of the sympathetic system promotes choroidal vasoconstriction in a similar way as in the intrarenal vessels. Thus, choroidal microcirculation may reflect renal microcirculation, especially in diseases characterised by excessive activation of the sympathetic system, such as CKD [3]. On the other hand, retinal circulation, being autoregulated, is compared to cerebral circulation [26].

Patients with end-stage renal disease subjected to dialysis are at significant risk of systemic microcirculatory dysfunction [27].

The currently available optical coherence tomography (OCT) and OCT-angiography (OCT-A) examinations constitute a safe, quick, and repeatable method of assessing both the CT and the condition of ocular microcirculation. Importantly, they do not require contrast administration, which is crucial in patients with renal failure [28].

Previous studies have demonstrated that HD is associated with repetitive subclinical myocardial ischemia that is associated with microcirculatory dysfunction, which probably contributes to the increased risk of cardiovascular events in this group [29]. Studies also indicate decreased cerebral flow as a result of HD, as well as cerebral small vessel disease, and increased risk of stroke in patients subjected to HD [30]. Decreased ocular blood flow during HD is also associated with the risk of ischaemic conditions within the eye [15].

A simple non-invasive method of assessing the microcirculatory status and response, provided by modern imaging methods, such as OCT and OCT-A, is of significant clinical value. It enables a better understanding of the response of the choroid, as well as retinal and choroidal microcirculation, to changes occurring in the course of HD. It might also provide the possibility of indirectly assessing the functions of the autonomic nervous system, which would be very promising [16].

In this work, we present a review of the literature on the effect of HD sessions on CT and retinal and choroidal microcirculation.

## 2. Material and Methods

In September 2023, an extensive manual search was made through the major electronic databases (PubMed, Google Scholar) in order to identify relevant studies published on the effect of HD sessions on CT and retinal and choroidal microcirculation. In the search criteria, original articles and literature reviews from 2013–2023 were marked. The following search terms were used: ‘choroidal thickness’, ‘haemodialysis’, ‘optical coherence tomography’, and ‘optical coherence tomography angiography’, in different combinations. After compiling a list of potentially relevant articles, the full text of each paper was appraised, with particular emphasis on articles presenting the impact of HD sessions on CT and retinal and choroidal microcirculation. A total of 22 compatible research publications were identified and used to compile this review.

## 3. Discussion

The impact of HD on the eye microcirculation system has been of interest to researchers for a long time [31,32,33]. Nowadays, the development of imaging techniques provides the possibility of the broader assessment of microcirculatory response to stress stimuli associated with HD sessions [4,5,6,7,8,9,10,11,12,13,14,15,16,17,18,19,20,21,22,23,24].

The eye includes two vascular compartments with a totally different flow regulation, which makes the assessment of ocular microcirculation so much more attractive [34].

The choroidal circulation is controlled by the extrinsic autonomic innervation. The reduction in choroidal blood flow occurs through the activation of the efferent sympathetic nerves, which release noradrenaline, thus activating the alpha 1-adrenergic receptors on the vascular smooth muscle cells. In turn, the increase in choroidal blood flow is mediated by parasympathetic efferent nerves, which act through nitric oxide signalling. The choroid is also densely innervated by the sensory fibres of the trigeminal nerve. There are also reports of some degree of choroidal blood flow autoregulation, but this issue requires further studies [34,35].

The retina tends to maintain a constant blood flow, despite changes in perfusion pressure, blood gas tension, and IOP. It is an internal autoregulatory function because the potential influence of autonomic innervation may be excluded [34,35].

The possibility of assessing CT seemed very attractive, especially due to the assumption that choroidal blood flow is poorly autoregulated and the ocular blood flow is an important factor affecting CT [9]. However, the correlation between CT and blood flow does not seem to be entirely obvious [34,35,36]. Moreover, since the choroid is composed of various tissues, such as blood vessels, nerves, and connective tissue, measuring the CT alone does not provide us with information on which tissues in the choroid change [11]. The CT changes may be caused by blood vessels, stroma, or both of these factors. Additionally, it has been indicated that CT alone may not be an accurate tool in clinical trials because many ocular and systemic factors influence CT [37]. Therefore, studies have attempted to assess the choroidal vascularity index (CVI), a new marker for the assessment of the choroid, which was also assessed in patients before and after HD [12,17].

Recently, the occurrence of OCT-A, which allows non-invasive identification of various retinal vascular plexuses and choroidal blood perfusion, has enabled even better assessment of the ocular microcirculatory response of CKD patients to HD sessions [16,17,18,19,28].

When assessing the microcirculatory response to HD, it should be considered that CKD patients undergoing HD already demonstrate visible changes in the microcirculatory status and autonomic system functions, and the degree of these changes may affect the variable response regarding selected parameters such as RT and CT, CVI, or vessel density (VD) [38].

The available studies also show that patients with CKD have a thinner retina and choroid, as well as reduced superficial capillary plexus (SCP) and deep capillary plexus (DCP) VD [39]. Basiony et al. demonstrated that higher stages of CKD were associated with a thinner choroid and retina. The authors observed that CRP and albumin were inversely correlated with CT and constituted independent predictors of this value. The authors point out that these findings can be explained by the role of inflammation in the development of vascular and kidney diseases [40]. In the study by Balmforth et al., a thinner choroid was associated with a lower estimated glomerular filtration rate (eGFR) and, in the case of CKD, proteinuria, as well as increased levels of circulating C-reactive protein, interleukin 6, asymmetric dimethylarginine (ADMA), and endothelin−1 [41].

It is suggested that the observed CT and RT changes in patients with CKD subjected to HD may be caused by disorders of the autonomic nervous system (ANS), which may be caused by the high level of inflammatory markers often observed in this group of patients [39,40]. While the choroidal circulation is controlled by autonomic innervation, the retinal circulation is not, so thinning of the outer retina and choroid may be related to increased sympathetic tone affecting the choroidal vessels [39].

Many pathological conditions underlying kidney damage (e.g., diabetes mellitus (DM), autoimmune disease) induce uraemia-related neuronal toxicity and thus affect the normal function of the ANS [42].

It should be kept in mind that ANS plays a key role in maintaining haemodynamic stability [43]. Therefore, the choroidal response to HD may differ depending on whether ANS dysfunction is present in a given case.

Studies regarding the impact of HD on CT and RT, or SCP, DCP, and choriocapillaris VD have produced conflicting outcomes (Table 1), which may result from significant differences in the methodology (Table 2) [4,5,6,7,8,9,10,11,12,13,14,15,16,17,18,19,20,21,22,23,24]. Studies differ with respect to such variables as number of patients examined, patients’ age, and gender [4,5,6,7,8,9,10,11,12,13,14,15,16,17,18,19,20,21,22,23,24]; in some studies, only men were included [8]. It is well known that CT depends on age and gender [44]. A significant majority of the studies concern Asian populations [4,9,10,11,12,14,15,18,19,20,22,23,24], and racial differences may play a significant role [45]. The study groups differ in terms of the assessed CKD aetiology, which is definitely influenced by ethnicity [46]. The studies also differed in terms of the OCT types used to perform them; some studies that assessed CT did not use the EDI-OCT function, which could limit the ability to accurately assess CT [4,22]. In some studies, the inclusion criteria did not take into account such parameters as axial length and refraction [4,5,6,11,18], which may influence the CT [47]. Some studies assessed the effect of HD directly before and after the HD session, while some provided information regarding the interdialytic interval [13,17]; in other studies, there was a two-week gap between the tests, and the results of which were being compared [11]. It is also worth considering the exclusion criteria—some studies excluded patients after eye surgery or who were smokers due to the influence of these factors on the CT [14]. The adjustment of the results for diurnal variation of the CT also differed between studies, and in some studies, the diurnal variation was not considered [4,10,12,14,18,20,23], despite the fact that it affects CT [48]. The studies also differ in terms of the time for which the patients have been subjected to HD, with single studies assessing patients undergoing their first HD session [11], which may also be of importance, as the duration of the HD may affect the microcirculatory response [38]. In most studies, the measurement was performed manually, although there were differences regarding the CT measuring point [4,5,6,7,8,9,10,11,12,13,14,15,16,17,18,19,20,21,22,23,24]. It is well known that CT differs depending on the place of measurement [49]. Finally, some studies included both eyes of the same patients, which might introduce potential bias by using linked outcomes [11,14,20,21,22].

Despite these many differences, the results of a meta-analysis performed by Su et al. demonstrated that HD caused a significant subfoveal CT (SCT) decrease, especially in patients with DM and proliferative diabetic retinopathy (PDR), which indicated that CKD aetiology determined the HD impact on CT. Furthermore, a sub-analysis revealed that adjusting for diurnal variation, using different types of OCT, or using different scanning modes, did not result in significant differences in the observed SCT changes [50]. Although most studies demonstrate a decrease in CT, the explanation of the mechanism of this decrease and the influence of systemic factors varies significantly between studies, and these differences are noteworthy.

A comparison of the ocular parameter changes during HD according to the original cause of HD was assessed in the study by Chen et al., which demonstrated that different CKD aetiologies tended to have the same overall trend on the ocular parameter changes during HD. In this study, the patients were divided into those with primary kidney disease, hypertensive kidney disease, diabetic kidney disease, and unknown aetiology, including patients with overlapping aetiologies. The subgroups are quite small, which significantly limits the value of the obtained results. Nevertheless, in the case of most parameters, the changes followed a similar trend, yet, lower best-corrected visual acuity, increased central corneal thickness, and decreased CT and RNFL thickness were observed in diabetic kidney disease [10].

In the study by Kang et al., DM was the only factor significantly correlated with SCT differences observed after HD sessions [24].

Due to the possible influence of DM on the results of CT measurements, some studies excluded patients with DM [5,7,8,15]; in other studies, we can find comparisons between patients with DM and without DM (non-DM) regarding CT changes observed as a result of HD session [6,9,10,11,12,14,18,19,23,24].

Both in the study by Kal et al. and in the study by Ulaş et al., as well as in the studies by Mayali et al. and Wang et al., which excluded patients with DM from the study group, the measurements performed after HD demonstrated a statistically significant thinner choroid in all measuring points compared to measurements before HD. The authors associated the reduced CT with ultrafiltration-induced hypovolemia and increased plasma colloid osmotic pressure and also noted that the lack of choroidal autoregulation might influence the observed changes in its thickness. [5,7,8,15].

In contrast to their results, Jung et al. demonstrated a statistically significant CT increase after HD. Unlike the above-mentioned studies, this study included patients with DM. In the study by Jung et al., the analysis of SD-OCT images showed an increase in SCT accompanied by an increase in its extravascular density. The authors assumed that the CT alteration may be mediated by choroidal autoregulatory control in response to hemodynamic changes [4].

In the context of the unclear extent of choroidal autoregulation, it is worth noting that vascular calcification and vasomotor dysfunction occur in the majority of HD patients as a result of systemic diseases and side effects of HD, impair the adaptive capacity of the vascular bed, and the autoregulatory response of the choroid, which occurred in this group, may differ from that in healthy people [15]. In the study by Zhang et al., the authors explained the lack of changes in CT to the impaired autoregulation ability of the choroidal tissue due to hypertension and DM often present in this group of patients with end-stage renal disease [18].

Some authors attribute the CT decrease to an ASN dysfunction characterised by the predominance of the sympathetic system activity [6,14,15]. The renin–angiotensin–aldosterone system and sympathetic system may be activated due to hypovolemia caused by ultrafiltration. It should be kept in mind that all the components of the renin–angiotensin–aldosterone system are extensively expressed in the vascular networks in the retina and choroid. Similar to what happens in the kidneys, angiotensin II, acting through the type I receptors, causes the constriction of retinal and choroidal vessels [3]. The authors assumed that the mechanism of an SCT decrease may result from the stronger constriction of the vascular smooth muscles and non-vascular smooth muscles in the choroid due to the activation of the sympathetic ANS [14].

However, in the study by Ishibazawa et al., multiple regression analysis did not demonstrate a significant correlation between changes in CT and changes in BP and heart rate in the two groups, i.e., DM and non-DM patients. Thus, the authors assume that the CT changes they observed were not significantly influenced by autonomic choroidal control. The authors of the study attributed the reduction in CT to the removal of intravascular and interstitial fluid due to the increased transcapillary colloidal osmotic gradient [22].

Ulaş et al. indicated that they had not observed intradialytic hypertension, which would indicate excessive activity of the sympathetic nervous system; therefore, they concluded that the muscular mechanism did not play a significant role in the process of choroidal thinning after HD, which they associated with hypovolemia caused by ultrafiltration and increased colloid osmotic pressure [8].

The assessment of the ANS effect on CT still requires further studies giving special consideration to the evaluation of the ANS function in CKD patients subjected to HD.

### 3.1. Choroidal Thickness Changes Due to HD in Patients with and without Diabetes Mellitus

Also, studies comparing the CT changes before and after HD in DM and NDM patients bring conflicting results [6,9,10,11,12,14,18,22,23,24].

In the study by Çelikay et al., mean SCT after HD decreased significantly from 254.59 ± 84.66 µm to 229.34 ± 77.79 µm (*p* < 0.001); there was no difference in CT between patients with and without DM before and after HD [6]. In the study by Shin et al., in which OCT-A was used, no statistically significant differences were observed in the comparison of changes in selected OCT-A parameters between patients with and without DM in response to HD treatment [19]. In the study by Zhang et al., VD of the outer retina after HD decreased statistically significantly in the DM and non-DM groups [18].

In the study by Kang et al., the mean ratio of SFCT change did not differ significantly between the groups, but mean SCT differences were significantly larger in patients with DM compared to non-DM patients [24].

Major CT changes in DM patients may result from the presence of choroidopathy. Choroidopathy in patients with DM includes choroidal occlusion and/or atrophy, increased formation of vascular loops, microaneurysms, vascular remodelling, intrachoroidal neovascularisation, and, as a consequence, choroidal circulation disturbance [51]. Moreover, the presence of diabetic choroidopathy may affect the autonomic choroidal compensation mechanism [23]. Due to the above, the choroidal response to HD in DM patients may be different, as indicated by some studies [10,11,22,23,24].

In the study by Chang et al., CT after HD decreased in all areas (*p* < 0.001). In the group of DM patients, mean CT changes were greater than in non-DM patients (*p* < 0.05). The authors assumed that the observed changes resulted from the occurrence of vascular alterations in the choroid associated with DM and the higher decrease of serum osmolarity and weight loss in DM patients. Chang et al. subdivided the DM patients into two groups: with severe retinal changes and with moderate retinal changes. The severe retinal change group showed greater CT alterations in all areas [23]. Similar results were obtained in other studies [22].

The study by Ishibazawa et al. was the first one to demonstrate that CT changes were more pronounced in DM. The reduction in CT was observed in the eyes of all patients with DM, but greater changes were noted in patients after panretinal photocoagulation (PRP), the reason for the greater decrease in SCT after HD in PRP eyes may not only be associated with the severity of the diabetic choroidopathy but also with the possibility of the influence of PRP itself [22]. However, the changes in choroidal circulation after PRP remain controversial [52].

### 3.2. Correlation between the Choroidal Thickness Changes and the Selected Systemic Parameter Changes

Regardless of the aetiology of CKD, changes in systemic parameters observed during HD sessions may have a significant impact on the CT. However, the degree of these changes differs in particular studies (Table 3), which may affect the obtained results regarding CT.

In most studies, we find references to changes in systolic BP (SBP) and diastolic BP (DBP), while some studies assessed mean arterial pressure (MAP), body weight, and body fluid removal (BFR), while other studies included references to plasma colloid osmotic pressure, serum osmolarity, or ocular perfusion pressure (OPP) [4,5,6,7,8,9,10,11,12,13,14,15,16,17,18,19,20,21,22,23,24]. The results of studies regarding the correlation between the changes in systemic parameters and CT changes differ in respective studies [4,6,8,9,12,13,14,15,18,19,22,23,24]. There are studies that did not demonstrate any influence of the selected systemic parameters, including BP changes, body weight changes, and BFR on the CT [12,13], and ones that indicated a correlation between selected systemic parameters and CT [4,6,9,14,15,19,22,23] (Table 4). Some studies associated the SCT reduction with a DBP decrease only [6]. In other studies, the decrease in CT was associated with BFR only [22].

### 3.3. Ultrafiltration

The factor responsible for a series of significant systemic changes in the course of HD is ultrafiltration. During an HD session, ultrafiltration removes excess fluid from the plasma, which leads to a loss of blood volume, an increase in plasma protein concentration (increase in the plasma colloid osmotic pressure), and a decrease in serum osmolarity [53]. The ultrafiltration volume is responsible for the amount of fluid removed during HD [12]. Some studies did not demonstrate a correlation between ultrafiltration volume and CT change [8,13,14]. Other studies, in turn, demonstrated a correlation between differences in CT and the amount of ultrafiltration [15].

In the study by Shin et al., ultrafiltration volume was significantly correlated with the central and total CT changes in the entire group of patients. The ultrafiltration volume did not differ significantly between DM and non-DM patients. However, considering the subgroups, only in non-DM patients, a significant correlation was found between ultrafiltration volume and central/total CT changes. No correlation was found between changes in choriocapillaris-perfused vessel density and ultrafiltration volume in patients with and without DM [19].

In the study by Ishibazawa et al., the mean BFR in the DM group was significantly higher than that in the non-DM group, and therefore, delta SCT/BFR was calculated to assess choroidal changes per unit change in the body fluids. The delta SCT/BFR value in the DM group was significantly higher than that in the non-DM group. However, the linear regression analysis demonstrated that the correlation between delta SCT and BFR was not significant in the DM group, but it was significant in the non-DM group. In the multiple regression analysis, BFR was independently correlated with delta SCT only in the non-DM group [22]. However, this study did not assess which component, the vascular or interstitial system, underwent greater changes in the choroid [22]. Recently, Sonoda et al. demonstrated that the areas of the choroidal vascular lumen and interstitium may be analysed separately using image binarization through SD-OCT [54].

In the study by Nakano et al., the image binarization technique was used, which enabled them to assess the changes in stromal and luminal areas. In both groups, the DM and non-DM patients, there was a statistically significant reduction in CT and large choroidal vessel layer thickness compared to choriocapillaris-medium choroidal vessel layer thickness, which did not change significantly in either group. The comparison of each layer change ratio between the DM and non-DM patients demonstrated a statistically significantly higher SCT reduction, as well as a large choroidal vessel layer thickness in the DM group. In both the DM and non-DM groups, there was a statistically significant decrease in the total choroidal area (TCA) and luminal area (LA). The stromal area (SA) decreased in the DM group and the non-DM group, but the results were not statistically significant. The comparison of changes in the LA and TCA in the DM and non-DM patients demonstrated greater statistically significant changes in the DM group [11].

Shin et al. also used image binarization and determined the LA, SA, TCA, SCT, and CVI. Total CT demonstrated a significant overall decrease after HD (−10.9 ± 14.0, *p* < 0.001). In the subgroup analysis, it was found that total CT decreased significantly in patients with DM (−11.3 ± 13.6, *p* = 0.004) and non-DM (−10.6 ± 14.9, *p* = 0.020), but the CT reduction was observed in more subfields in patients with DM than those without. With the CT reduction, both choroidal LA and SA also decreased at the same time, while CVI did not demonstrate significant change after HD. Moreover, no significant difference was observed between DM and non-DM patients in LA, SA, TCA, and CVI before and after HD [12]. In the choroid, the vascular system is well fenestrated, and the extravascular (interstitial) compartment is characterised by a very high permeability of fluids and small particles. The decreased CT, LA, and SA after HD may be associated with the removal of intravascular and interstitial fluids due to an increased transcapillary colloid osmotic gradient. The authors hypothesized that histological changes and the impairment of the ANS in the diabetic choroid may lead to dysregulation of both osmotic and hydrostatic pressure and that greater changes in the parameters assessing the condition of the choroid can be expected after HD in the DM group [12]. Also, Nakano et al. point out that the increased permeability of the choroid may be significant both before and after the initiation of HD in the DM group due to diabetic choroidopathy [11]. It is worth noting that HD increases oxidative stress, and the circulatory levels of various endogenous vasoactive substances can easily pass from the fenestrated choriocapillaris to the interstitial area, causing severe vasoconstriction [55,56].

Jung et al. observed CT and extravascular density increase. In this study, the authors also pointed out that the changes in the choroidal density in OCT images resulted from high choroidal vascular permeability. As a result, the outflow of macroparticles, such as albumin, and small particles, such as glucose, was to the extravascular space. According to the authors, if these particles are characterised by high optical dispersion properties, the extravascular choroidal density may change after HD [4].

### 3.4. Blood Pressure

In most studies performed in patients after HD, an SBP decrease was observed after an HD session; however, the impact of these changes on CT differed between respective studies [4,6,8,12,19,23,24]. Chang et al. observed a significant correlation between SBP and SCT change [23]. Elbay et al. determined SCT during HD. Mean SCT decreased significantly from 270.85 ± 73.82 µm before HD to 257.01 ± 71.49 µm in the second hour of HD (*p* < 0.001). After an HD session, it increased slightly to 258.44 ± 75.17 µm, but it was still significantly lower than before the HD session (*p* < 0.001). Systolic blood pressure decreased significantly from 140 (80–180) mmHg before HD to 120 (50–170) mmHg in the second hour of HD (*p* < 0.01) and 120 (80–260) mmHg after the HD session (*p* < 0.001). No correlation was found between the SCT percentage changes and systemic parameters, including SBP [13]. Similarly, in the study by Zhang et al., there was no correlation between the change in SBP and DBP and the changes in SCT after HD [18].

In the study by Sun et al., the SCT difference was correlated only with baseline SBP, but there was no correlation with SBP change, DBP change, or baseline DBP [14].

Jung et al. suggested that the increased SCT after HD was significantly negatively correlated with SBP and that the increase was due to choroidal vasodilation induced by the choroidal autoregulatory response to the reduction in blood pressure [4].

In the study by Shin et al., the reduction in the choriocapillaris-perfused VD was associated with the BP decrease, including SBP and MAP [19]. Ulaş et al. also determined the MAP, but they did not find a correlation between the SCT difference and the MAP difference [8].

In the study by Çelikay et al., SBP, DBP, and SCT decreased after HD. Although there was no correlation between the changes in SCT and SBP changes, there was a positive correlation between the changes in SFCT and DBP changes. Choroidal thickness change was mostly correlated with DBP change, which suggests that the CT may be associated with changes in BP [6]. In the multivariate analysis, only the change in DBP explained the decrease in CT; the authors suggest that the decrease in DBP associated with intradialytic hypotension (IDH) leads to the activation of the sympathetic nervous system to avoid hypotension, and this leads to a decrease in CT [6].

Both the rate of ultrafiltration and the degree of volume replacement from the interstitial compartment during HD may result in IDH, defined as a relative or absolute decrease in BP, as well as the occurrence of specific symptoms. Patients subjected to HD may not tolerate such a high decrease in blood volume as healthy people. The presence of a heart condition and autonomic neuropathy may exacerbate IDH and impair peripheral blood circulation. Interdialytic hypotonia and, subsequently, organ hypoperfusion, may lead to multi-organ ischaemia. Apart from the cardiac muscle, it affects the brain and intestines and may lead to the occurrence of infarction in these organs and their permanent dysfunction [57]. Coppolino et al. showed that a simple measurement of retinal and choroidal parameters using OCT-A before an HD session may help predict the short-term risk of the occurrence of IDH [16].

### 3.5. Body Weight

The correlation between CT and body weight change after HD differs in various studies [4,6,9,12,13,14,19,23,24]. In the study by Jung et al., body weight loss was associated with an SCT increase, but this correlation failed to reach statistical significance [4]. In the study by Yang et al., CT reduction was correlated with the body weight reduction [9]. Similar results were obtained by Sun et al. In their study, body weight change was correlated with CT change [14]. Chang et al. also demonstrated that body weight loss was correlated with CT change [23]. On the other hand, in studies by Çelikay et al., body weight decreased statistically significantly after HD, but the weight change was not correlated with CT change [6]. In the study by Elbay et al., such a correlation was not demonstrated either [13].

### 3.6. Plasma Colloid Osmotic Pressure and Serum Osmolarity

Changes in plasma osmolarity determine fluid transfer between intracellular and extracellular compartments. It should be kept in mind that the extent of hypovolemia depends on the balance between the rate of fluid removal and vascular refilling, and this, in turn, is associated with colloid osmotic pressure value. The relative increase in colloid osmotic pressure caused by water removal during HD generates a colloidal osmotic pressure gradient between the plasma and interstitial fluid, causing water to move from the interstitial fluid to the plasma [58]. Colloid osmotic pressure is the main factor affecting fluid transfer between the interstitial and vascular compartments. This may lead to a decrease in CT due to fluid removal from blood vessels and choroidal stroma after a single session of HD [13].

However, not all studies assessed the effect of plasma colloid pressure [4,8,15,22,23,24]. The ones that did evaluate this parameter indicated its increase [11] or even reduction following HD [15].

However, in the study by Ulaş et al., as well as the study by Çelikay et al., no significant correlations were found between plasma/serum osmolarity and SCT change [6,8]. In contrast to the above results, Chang et al. demonstrated a significant correlation between serum osmolarity changes and CT changes. A greater change was observed in patients with DM [23].

In the study by Jung, colloid osmotic pressure increase and serum osmolarity decrease were marginally associated with an SCT increase [4].

It should be borne in mind that, due to individual differences, the time of plasma osmotic pressure recovery varies in individual patients; in some patients, plasma osmotic pressure returns to pre-HD levels soon after the completion of HD, which may also explain the discrepancies between the results of studies that assessed the relationship between this parameter and CT [15].

### 3.7. Ocular Perfusion Pressure

Ocular perfusion pressure represents the pressure of blood flowing in the blood vessels in the eye and is defined as the difference between MAP and IOP, which is an important factor regulating blood flow in the eye [15]. MAP measurements before and after HD, as well as IOP measurements before and after HD, differed in the reviewed studies, which finally translates into varied OPP results [4,5,11,15,18,22,23].

In the study by Kal et al., the SBP, DBP, and MAP decreased statistically significantly, IOP decreased but not statistically significantly, OPP decreased statistically significantly, and CT was reduced, but no correlation with systemic variables and OPP was assessed [5]. The study by Ishibazawa et al. showed no significant changes in IOP, a significant decrease was noted in relation to SBP, and finally, OPP decreased statistically significantly in both DM and NDM patients, but no correlation was found between OPP changes and CT changes [22]. Similarly, in the study by Zhang et al., a statistically significant OPP decrease was observed in both the DM and NDM groups [18].

In the study by Wang et al., OPP, SBP, DBP, and IOP were assessed before, at 2 h, and after the completion of an HD session. OPP tended to decrease from the beginning of the session until two hours after HD but subsequently returned to the pre-HD level. In this study, a similar trend was observed for SBP, but IOP did not demonstrate statistically significant changes during the measurements. HD caused a transitional OPP reduction, which may contribute to the occurrence of ischaemic conditions [15].

It should also be taken into account that there is a correlation between IOP and CT; the choroid modulates IOP through vasomotor control of the blood flow and aqueous humour outflow from the anterior chamber through the uveoscleral route [13,35]. With regard to IOP changes before and after HD, the studies demonstrated highly varied results, the discussion of which goes beyond the scope of this work.

However, in the context of the impact of IOP and its changes on OPP and CT, it is worth paying attention to the study by Mayali et al., which assessed the ocular pulse amplitude (OPA) [7], defined as the difference between the minimum (or diastolic) and maximum (or systolic) values of the pulsatile IOP [59]. OPA is considered an objective parameter reflecting choroidal perfusion [60].

The mean OPA values before and after HD were 2.14 ± 1.07 (0.6–4) mmHg and 1.6 ± 0.86 (0.5–3.2) mmHg (*p* < 0.001), respectively. A statistically significant correlation was found between OPA measurements and CT (*p* < 0.001, R = 0.923). IOP increased from 15.11 ± 2.58 (11–20) to 15.99 ± 2.21 (13–20) mmHg, but the change was not statistically significant (*p* = 0.05). The authors explained that the decrease in OPA was consistent with decreased choroidal blood flow due to ultrafiltration-induced hypovolemia, increased plasma colloid osmotic pressure, and decreased systolic blood pressure. Moreover, in this study, the authors pointed out that a decrease in SBP could lead to sympathetic nervous system activation caused by hypotension, which may affect the CT [7].

Interestingly, systemic hypotension does not activate sympathetic input to the choroid but does cause peripheral vasoconstriction; high BP drives choroidal vasoconstriction, which indicates that there is a difference between inputs to the central neurons giving rise to the sympathetic postganglionic outflow to the systemic versus the choroidal vasculature [35].

### 3.8. Other Factors

The electrolyte and calcium-phosphorus metabolism alteration that occurs during HD may also affect CT. Chang et al. found a statistically significant decrease in sodium and potassium after HD, but it was not assessed whether these changes were related to changes in CT [23]. Several studies referred to such parameters as sodium, potassium, calcium, parathyroid hormone, and phosphorus [10,21,23]. The study by Chen et al. demonstrated that the changes in CT were negatively correlated with sodium levels, while the changes in average RT were positively correlated with potassium levels before HD [10]. The issue of changes in electrolyte concentration and their impact on the CT, as well as the impact of changes in partial concentrations of oxygen and CO2 occurring during HD on the CT, require evaluation in subsequent studies. It should be kept in mind that choroidal vessels are susceptible to partial gas pressure changes. Although there is a general consensus that blood gases (pO2 and pCO2) control retinal blood flow, the choroidal circulation is largely insensitive to changes in pO2. However, the increase in pCO2 elevates the choroidal blood flow in humans [34,35].

A very interesting issue is the impact of changes in cytokine levels that occur as a result of HD on the CT, also in the context of its impact on the ANS; this topic has not been discussed in the literature yet. Another factor that may have a significant impact on choroidal blood flow is the patient’s temperature because during HD, the temperature of the dialysate causes a drop in body temperature, which may affect the change in choroidal blood flow [21,35].

### 3.9. Retinal Thickness

While discussing the changes in microcirculation observed as a result of HD, we cannot omit the reaction of the retina to the HD procedure. On the one hand, blood flow in the retina is autoregulated and should, therefore, remain constant despite changes occurring during HD. On the other hand, it should be taken into account that the retina is supplied by the central retinal artery (2/3) and by the choroidal circulation (1/3); therefore, it is interesting to assess whether the CT reduction affects the RT [18,61].

In many studies, RT measured before and after HD remained stable [8,9,10,11,16,23]. However, in some studies comparing the RT in DM and non-DM patients, significant differences were found between the groups [9,14,23]. In the study by Yang et al., the central foveal thickness in the non-DM group increased, while in the DM group, it decreased; in the case of macular volume, there was an increase in the non-DM patients and a decrease in the DM patients; however, no statistical significance was established here. The authors indicated that the observed changes might be due to retinal flow autoregulation, but in the case of blood-retinal barrier damage in DM, this mechanism is not fully understood [9]. In the study by Shin et al., no statistically significant differences in RT changes were observed between DM and non-DM patients [19]. In the study by Zhang et al., after HD, RT was not statistically significantly lower in either of the two groups; however, a statistically significant decrease in RT after HD was observed in the entire study group [18]. In this study, SCP and DCP VD did not demonstrate a statistically significant change; a statistically significant decrease in VD was observed in the outer retina in both the DM and non-DM groups. A VD decrease in the outer retina was consistent with changes in RT [18]. In contrast to these results, the study by Wang et al. showed a statistically significant increase in RT at three measuring points (fovea, nasal, and temporal). The authors of the study concluded that as a result of HD, the retinal autoregulation capability might cause oedema [15]. Similarly, in the study by Chen et al., there was a trend towards an RT increase; in a few subfields, there was a statistically significant RT increase. This study also demonstrated a statistically significant increase in RNFL thickness. The authors attributed these changes to a decrease in plasma osmotic pressure, with liquid moving into the layers of the retina according to the gradient, leading to retinal oedema [10]. In the study by Hwang et al., which only included DM patients after the initiation of dialysis, the incidence of any macular oedema significantly decreased. Changes in serum osmolality after a dialysis session in DM patients may affect RT, which, according to the authors, may ultimately reduce macular oedema [20].

In the study by Sun et al., central macular thickness did not undergo statistically significant changes in the entire study population, a statistically significant decrease was only observed in DM patients. In this study, the retinal arterial caliber (RAC) and retinal venous caliber (RVC) were assessed, and it was found that they increased significantly after HD. RVC changes before and after dialysis were correlated with DBP, LDLC, and HDLC, as well as changes in body weight and DBP. RAC changes were not correlated with any systemic parameters. The mechanism responsible for vasodilation in HD is not clear. The authors noted that HD might lead to the removal of a nitric oxide synthase inhibitor, namely ADMA, which increases nitric oxide availability [14].

In the study by Coppolino et al., no statistically significant changes regarding CRT were observed before and after HD [16].

### 3.10. OCT-A

So far, there have only been a few studies assessing not only the RT and CT but also changes in angio-OCT parameters, such as VD in the SCP, DCP, outer retina, choriocapillaris, and FAZ before and after an HD session. These studies also differed in terms of the obtained results regarding the above parameters [16,17,18,19].

The study by Zhang et al. demonstrated a statistically significant decrease in the RT, VD of the outer retina, and OPP in the entire group as a result of an HD session. SCT, SCP/DCP VD, and choriocapillaris VD did not demonstrate statistically significant changes [18].

The study by Shin et al., unlike the study by Zhang et al., demonstrated a statistically significant change in the CT, which decreased, while the RT did not change significantly. Similar to the study by Zhang et al., VD in the SCP and DCP did not undergo significant changes, while total VD in the choriocapillaris decreased significantly after HD; however, no significant correlation was found between VD change in the choriocapillaris and CT change [18,19].

The above-described differences in the results of studies by Zhang et al. and Shin et al. may result from differences in the OCT-A devices applied in the respective study (SD-OCTA vs. SS-OCTA) or study protocol differences (3 × 3 mm vs. 6 × 6 mm) [18,19]. Furthermore, Shin et al. did not assess the outer retinal vascular density because, if there was no retinal pathology, OCT-A did not reveal a visible flow in the outer retina [19].

Regardless of the differences in the study results, the conclusion is that OCT-A may be an effective method for assessing microcirculation in HD patients.

The importance of OCT-A application in HD patients was demonstrated by Coppolino et al. [16]. The authors demonstrated that certain baseline OCT-A parameters significantly correlated with the occurrence of IDH episodes [16]. In the study by Coppolino et al., the analysis involved 35 eyes. Most of the analysed parameters remained unchanged after the completion of dialysis, and significant reductions were only observed in the case of central CT, 6×6 whole SCP VD, and 6×6 foveal DCP VD. Within a 1-month follow-up, a total of 73 IDH episodes were reported. The baseline OCT-A performed in patients experiencing IDH demonstrated statistically significantly thinner choroid and reduced FAZ-SCP 6 × 6, but larger 3 × 3 and 6 × 6 fovea-SCP VD, as well as larger 3 × 3 and 6 × 6 fovea-DCP VD. Of note, subjects experiencing IDH were older and had significantly lower SBP, DBP, and LDL cholesterol, but higher serum ferritin levels compared to subjects without hypotensive episodes, which could also contribute to the differences in central CT between these groups. In this study, a statistically significant positive correlation was observed between IDH and baseline foveal VD of SCP and DCP while an inverse correlation was found for central CT. Central CT, 3 × 3 foveal SCP VD, 3 × 3 and 6 × 6 foveal DCP VD, and 6×6 foveal SCP VD were strongly correlated with a higher risk of IDH within the 30-day follow-up. Moreover, it was determined that a CT below ≤ 242 was characterised by 100% test specificity and 47.3% test sensitivity in IDH prediction [16].

In another study, Coppolino et al. presented the results of an analysis of 23 eyes performed in HD patients, dividing them into those who experienced IDH episodes during the monthly follow-up and those without IDH episodes. Ten patients were included in the IDH group, and five were included in the no IDH group. The results of this study concerned not only the assessment of parameters before and after HD but were also enriched with data regarding changes in OCT-A parameters during the first hour, the second hour, and the third hour of an HD session. In the entire group, a significant decrease was only observed in DCP VD (total, foveal, and parafoveal) at the end compared to the beginning of an HD session and in central CT (*p* < 0.05). The general tendency observed during an HD session demonstrated a statistically significant decrease, especially in DCP VD (total, foveal, and parafoveal) and central CT (*p* = 0.006). In the group with IDH, SCP VD (foveal and parafoveal) increased significantly when comparing the values before to the ones after HD, while central CT decreased significantly. During the HD session, only central CT maintained a significant trend. In the group without IDH, the comparison of the values before and after HD, as well as a trend analysis, did not demonstrate statistical significance [17].

Although in both studies the CT decreased statistically significantly in the entire group, the choroidal response to HD in the IDH group differed between the studies [16,17]. In the 2021 study, the CT in the IDH group was significantly thinner than in the non-IDH group; moreover, a smaller thickness reduction was observed in the IDH group compared to the non-IDH group, which was believed to indicate impaired autonomic function [16]. The authors pointed out that, as a result of the Bezold-Jarisch reflex, the patient experienced a dramatic decrease in the activity of the sympathetic nervous system, accompanied by arterial vasodilation, bradycardia, and IDH. Whether this mechanism could be responsible for the smaller decrease in CT after HD in the IDH group is unclear, although such a mechanism is interesting, especially considering the fact that this reflex is conducted by the 5-HT receptors present in the choroid [16,62]. Nevertheless, the recurrent IDH episodes are associated with the risk of ischemia and, therefore, the CT in this group may be smaller, as shown by the authors; moreover, the occurrence of IDH episodes indicates impaired adaptive mechanisms in response to hypotension; thus, a smaller decrease in CT in this group may result from damaged adaptation mechanisms, or, for example, increased vascular wall stiffness.

However, in the 2022 study, contrary to the previous study, there was higher CT in the IDH group before HD; moreover, in the IDH group, a greater reduction in CT was observed. The results of these studies require re-evaluation in subsequent studies on larger groups of subjects; however, the usefulness of using OCT-A in clinical practice demonstrated in both studies is invaluable and opens a new area of research [16,17].

## 4. Conclusions

The development of imaging methods such as OCT and OCT-A provides an invaluable opportunity to assess microcirculation and understand the pathophysiology of systemic vascular diseases. Measurements of CT, CVI, and VD in retinal plexuses and choriocapillaris in patients with CKD undergoing HD expand our knowledge of the microcirculation status, as well as its response to stress related to a number of changes in systemic parameters related to the HD procedure. However, the currently available results are very diverse, and there are insufficient data for a full and reliable assessment of the correlation between the systemic changes that occur during HD and the CT or blood flow assessed using OCT-A. These limitations are not only related to the fact that the CT is affected by many variables but also to the fact that we are not fully aware of the physiological reactions of the choroid, especially in the context of blood flow autoregulation. There is no doubt that the assessment of autonomic function in HD patients using the choroidal response to HD-related stress is a very promising direction of research, but it requires further well-designed studies with an independent assessment of autonomic function in the study group. Finding the markers of ANS dysfunction in a simple, repeatable, and non-invasive test would be an invaluable advance in cardiovascular risk stratification in this group. Similarly, the correlation between CT, as well as choroidal/retinal microcirculation, and inflammation and endothelial dysfunction in response to HD warrant further studies.

## Figures and Tables

**Table 1:** Changes in choroidal thickness, retinal thickness, vessel density of the superficial capillary plexus, deep capillary plexus, outer retina and choriocapillaris, and foveal avascular zone in the entire group of the included studies.

Studies	Choroidal Thickness (µm) Mean ± SD		Retinal Thickness (µm) Mean ± SD		VD of the SCP	VD of the DCP	VD of the Outer Retina	VD of the Choriocapillaris	FAZ SCP
Jung et al. [4]	SFCT Before: 276.94 ± 58.73 After: 288.29 ± 65.57 *p* = 0.003		-		-	-	-	-	-
Kal et al. [5]	SFCT Before: median 182, range (103–374) After: median 161, range (90–353) *p* < 0.001 Significant decrease in all measured points		Before: median 246, range (179–296) After: median 248, range (141–299) *p* = 0.12		-	-	-	-	-
Çelikay et al. [6]	SFCT Before: 254.59 ± 84.66 After: 229.34 ± 77.79 *p* < 0.001 Significant decrease in all measured points		-		-	-	-	-	-
Mayali et al. [7]	SFCT Before: 255.21 ± 6.15 After: 234.95 ± 7.89 *p* < 0.001 Significant decrease in all measured points		-		-	-	-	-	-
Ulaş et al. [8]	SFCT Before: 232.81 After: 210.9 *p* < 0.001 Significant decrease in all measured points		Before:215.86 After: 216.9 *p* = 0.411		-	-	-	-	-
Yang et al. [9]	average choroidal thickness Before: 233.1 ± 77.5 After: 219.1 ± 76.8 *p* = 0.000		Before: 214.0 ± 21 After: 213.8 ± 21.8 *p* = 0.821		-	-	-	-	-
Chen et al. [10]	average choroidal thickness Before:289.55 ± 11.385 After: 254.134 ± 11.46 *p* < 0.001		Before:273.4 ± 3.302 After: 275.6 ± 3.18 *p* = 0.071 RNFL thickness Before:90.65 ± 1.829 After: 93.18 ± 1.974 *p* = 0.001		-	-	-	-	-
Nakano et al. [11] *	DM	NDM	DM	NDM	-	-	-	-	-
	SFCT Before: 301.7 ± 70.3 After: 261.6 ± 77.3 *p* < 0.001	SFCT Before: 281.8 ± 57.1 After: 259.8 ± 68.3 *p* < 0.001	Before: 277 ± 51.7 After: 255 ± 17 *p* > 0.05	Before: 276 ± 49 After: 255 ± 20 *p* > 0.05					
Shin et al. [12]	Total Before: 223.4 ± 78.3 After: 212.5 ± 81.6 Δ = 10.9 ± 14.0 *p* < 0.001 Central Before:232.8 ± 88.8 After:223.8 ± 93.8 Δ = 8.9 ± 22.3 *p* = 0.033		-		-	-	-	-	-
Elbay et al. [13]	SFCT Before: 270.85 ± 73.82 After the second hour: 257.01 ± 71.49 *p* < 0.001 After: 258.44 ± 75.17 it was still significantly lower than before haemodialysis *p* < 0.001		-		-	-	-	-	-
Sun et al. [14]	SFCT Before: 254.29 ± 69.36 After: 235.54 ± 59.9 *p* = 0.002		Before: 255.72 ± 35.29 After: 258.19 ± 48.26 *p* = 0.252		-	-	-	-	-
Wang et al. [15]	SFCT Before:267.97 ± 99.17 After: 255.87 ± 95.59 *p* < 0.01		Before: 207.88 ± 23.47 After: 209.94 ± 24.41 *p* < 0.05		-	-	-	-	-
Coppolino G et al. [16]	Δ Choroid central thickness 25 (10–57) significant reduction *p* < 0.001		Δ CRT 0 (− 3-2) *p* = 0.28		ΔWHOLE-SCP 3 × 3 mm 0.3 (− 1.6 − 1.7) *p* = 0.91 ΔWHOLE-SCP 6 × 6 mm 1.4 (0.6 − 3.5) *p* = 0.04 ΔPARAFOVEA-SCP 3 × 3 mm 0.5 (− 1.6 − 1.6) *p* = 0.8 ΔPARAFOVEA-SCP 6 × 6 mm 1 (− 0.8 − 3.3) *p* = 0.13 ΔFOVEA-SCP 3 × 3 mm − 0.5 (− 1.7 − 1.5) *p* = 0.12 ΔFOVEA-SCP 6 × 6 mm 0.5 (− 1 − 2.6) *p* = 0.82	ΔWHOLE-DCP 3 × 3 mm − 0.1 (− 3.2 − 1.2) *p* = 0.97 ΔWHOLE-DCP 6 × 6 mm − 0.6 (− 2.6 − 3.5) *p* = 0.26 ΔPARAFOVEA-DCP 3 × 3 mm 0.3 (− 3 − 1.9) *p* = 0.8 ΔPARAFOVEA-DCP 6×6 mm − 1.3 (− 3 − 4.7) *p* = 0.27 ΔFOVEA-DCP 3 × 3 mm − 0.1 (− 2.5 − 1.4) *p* = 0.26 ΔFOVEA-DCP 6 × 6 mm 2.5 (0.4 − 4.6) *p* = 0.02	-	-	ΔFAZ-SCP 3 × 3 mm 0 (− 0.01 − 0.01) *p* = 0.58 ΔFAZ-SCP 6 × 6 mm 0.05 (− 0.04 − 0.01) *p* = 0.11
Coppolino G et al. [17]	Central choroid thickness Before: 193.83 ± 43.88 After 1 h: 193 ± 47.62 After 2 h: 191.55 ± 46.14 After 3 h: 175.33 ± 49.00 After HD: 181.82 ± 47.77 *p* = 0.05 (before vs. after HD)		-		SCP Whole Before: 40.71 ± 5.13 After 1 h: 40.08 ± 5.78 After 2 h: 40.87 ± 5 After 3 h: 41.03 ± 5.16 After HD: 41.42 ± 3.14 *p* = 0.45 (before vs. after HD) SCP fovea: Before: 16.06 ± 6.85 After 1 h: 16.3 ± 7.33 After 2 h: 20.03 ± 8.75 After 3 h: 18.5 ± 7.48 After HD: 17.16 ± 7.03 *p* = 0.07 (before vs. after HD) SCP parafovea: Before: 43.4 ± 5.99 After 1 h: 42.67 ± 6.06 After 2 h: 42.08 ± 8.99 After 3 h: 43.89 ± 5.43 After HD: 44.08 ± 3.44 *p* = 0.5 (before vs. after HD)	DCP Whole Before: 47.76 ± 5.07 After 1 h: 46.72 ± 4.48 After 2 h: 47.64 ± 3.71 After 3 h: 46.83 ± 4.23 After HD: 42.82 ± 10.19 *p* = 0.02 (before vs. after HD) DCP fovea: Before: 32.26 ± 8.31 After 1 h: 31.60 ± 9.25 After 2 h: 33.54 ± 7.93 After 3 h: 33.69 ± 7.47 After HD: 30.59 ± 6.87 *p* = 0.03 (before vs. after HD) DCP parafovea: Before: 50.03 ± 5.44 After 1 h: 49.24 ± 4.50 After 2 h: 50.13 ± 3.67 After 3 h: 49.08 ± 4.77 After HD: 47.34 ± 3.84 *p* = 0.01 (before vs. after HD)	-	-	Before: 0.28 ± 0.11 After 1 h: 0.27 ± 0.11 After 2 h: 0.25 ± 0.11 After 3 h: 0.25 ± 0.12 After HD: 0.27 ± 0.12 *p* = 0.3 (before vs. after HD)
Zhang et al. [18]	SFCT Before:240.3 ± 91.7 After: 228.4 ± 82.3 *p* > 0.05		Before: 204.7 ± 22.4 After: 200.8 ± 22.8 *p* < 0.05		Before: 50.7 ± 3.1 After: 50.5 ± 3.2 *p* > 0.05	Before: 55.6 ± 2.5 After: 55.4 ± 2.5 *p* > 0.05	Before: 38.8 ± 5.5 After: 37.5 ± 3.4 *p* < 0.05	Before: 65.5 ± 1.7 After: 65.6 ± 1.5 *p* > 0.05	-
Shin YU et al. [19]	Total CT: Before: 209.6 ± 64.6 After: 195.2 ± 64.1 *p* < 0.001 Central CT: Before: 214.8 ± 80.9 After: 199.1 ± 83.2 *p* < 0.001		Total RT: Before: 267.3 ± 15.5 After: 267.2 ± 15.5 *p* = 0.866 Central RT: Before: 235.7 ± 40.6 After: 235.4 ± 41.5 *p* = 0.759		Total Before: 21.7 ± 6.3 After: 21.9 ± 5.7 *p*= 0.772 Central Before: 5.2 ± 5.6 After: 5.3 ± 3.8 *p*= 0.917	Total Before: 8.5 ± 5.7 After: 8.9 ± 4.8 *p*= 0.512 Central Before: 2 ± 3.2 After: 1.5 ± 1.8 *p*= 0.291	-	Total Before: 46.2 ± 11.3 After: 43.3 ± 10.7 *p* < 0.001 Central Before: 37.5 ± 14.3 After: 34.7 ± 14.1 *p* = 0.007	-
Hwang et al. [20]	SFCT Before: 313.31 ± 85.29 After: 288.81 ± 92.02 *p* = 0.001		Before: 317.92 ± 91.41 After: 287.77 ± 57.55 *p* = 0.024		-	-	-	-	
Ishibazawa et al. † [22]	DM	NDM	DM	NDM	-	-	-	-	-
	SFCT Before: 268 ± 75 After: 234 ± 69 *p* < 0.05	SFCT Before: 233 ± 78 After: 217 ± 72 *p* < 0.05	Before: 259 ± 40 After: 260 ± 33 *p* > 0.05	Before: 263 ± 29 After: 268 ± 28 *p* > 0.05					
Chang et al. [23]	SFCT: Before: 233.6 ± 45.2 After: 214.2 ± 43.8 *p* < 0.001 Significant decrease in all measured points		-		-	-	-	-	-
Kang et al. [24]	SFCT Before: 311.8 ± 64.9 After: 311.2 ± 65.1 *p* = 0.877		-		-	-	-	-	-

Abbreviations: CT, choroidal thickness; CRT, central retinal thickness; DCP, deep capillary plexus; DM, diabetes mellitus; FAZ, foveal avascular zone; NDM, non-diabetes mellitus; SCP, superficial capillary plexus; SD, standard deviation; SFCT, subfoveal choroidal thickness; RNFL, retinal nerve fiber layer; RT, retinal thickness; VD, vessel density. * SFCT changes were significantly greater in the DM group than in the NDM group. (−13.3 ± 2.5% vs. −9.5 ± 3.1%; *p* = 0.049). † SFCT changes were significantly greater in the DM group than in the NDM group. (−12.6 ± 3.4% vs. −6.9 ± 2.3%; *p* = 0.00027).

**Table 2:** Characteristics of the included studies.

Studies	Country	OCT Type	Number of Patients	Number of Eyes	Mean Age	Sex Ratio (Male/Female)	Mean HD Duration	Hemodialysis Parameters Blood Flow Rate/Dialysate Flow Rate (mL/min)	CT-Measured Areas	Ophthalmological Parameters Evaluated before and after HD	Patients Divided into DM and NDM Group
Jung et al. [4]	Korea	SD-OCT Heidelberg	19	28	51.21 ± 9.47	7/12	3.50 ± 1.50 years	250/-	SFCT	choroidal extravascular density SFCT IOP	No
Kal et al. [5]	Turkey	SD-OCT Optovue	25	-	-	17/8	42.8 months	250–300/500	SFCT; 500 μm and 1000 μm nasal to the fovea 500 μm, 1000 μm, and 1500 μm temporal to the fovea	CT, RT, IOP	No, Patients with DM were excluded from the study
Çelikay et al. [6]	Turkey	SD-OCT Optovue	41	41	53.2 ± 14.6	16/25	42.81 ± 30.38 months	250–300/500	SFCT and 1500 μm and 3000 μm nasal and temporal to the fovea	CT, IOP	Yes
Mayali et al. [7]	Turkey	SD-OCT Cirrus EDI mode	22	22	56.14 ± 9.96	8/14	56.7 ± 51.9 months	250/-	SFCT and at 1500 μm and 3000 μm nasal and temporal of the fovea	IOP, OPA, CT CCT, ACD, LT, AXL	No Patients with DM were excluded from the study
Ulaş et al. [8]	Turkey	SD-OCT Heidelberg	21	21	61.81	21	2.48 years	250–300/500	SFCT and 1500 μm nasal and temporal from the center of the fovea	IOP, CCT CT, RT	No Patients with DM were excluded from the study
Yang et al. [9]	Korea	EDI-OCT Heidelberg	34	34	58.2 ± 9.8	20/14	71.1 ± 60.8 months	250/-	Vertical and horizontal scans were taken through the fovea	CFT, CT, MV, RNFL thickness, IOP	Yes
Chen et al. [10]	China	Cirrus HD-OCT EDI mode	45	90	57.48 ± 13.57	27/18	70.09 ± 58.03 months	-/-	SFCT; 1000 μm and 2000 μm nasal and temporal away from the center of the macula, averaged, and recorded as the average thickness of the choroid.	TBUT, Schirmer’s I logMAR of BCVA, Spherical power, Cylinder power, RT, RNFL thickness, CT, corneal thickness IOP, ACD, LT, ECD, ECS, ECSCV	Yes
Nakano et al. [11]	Japan	SS-OCT Topcon	31	60	DM: 66.6 ± 10.6 NDM: 69.4± 10.7	16/15	-	-/-	average of all points in the inner circle (radius 1 mm) of the center of the nine sectors, which was defined by the ETDRS grid	CT, CMT choroidal vessel layer thickness, Choriocapillaris-medium choroidal vessel layer thickness, luminal area, stromal area, choroidal area, IOP, ACD, spherical equivalent	Yes
Shin et al. [12]	Korea	SS-OCT Topcon	32	32	56.4 ± 10.4	13/19	6.0 ± 4.1 years	250/500	Representative points within the ETDRS grid were determined as the central (fovea), and 1000 μm and 2250 μm temporal, superior, nasal, and inferior to the macula	IOP, AL, CT, CVI, LA, SA, TCA	Yes
Elbay et al. [13]	Turkey	SD-OCT Nidek	27	27	54.92 ± 18.84	16/11	3.37 ± 2.30 years	250/-	SFCT	SFCT, IOP, CCT, ICA	No
Sun et al. [14]	China	SD-OCT Optovue	202	404	54.76 ± 11.1	109/93	75.24 ± 66.04 months	200–300/500	SFCT	SFCT, CMT, RAC, RVC, IOP, BCVA	Yes
Wang et al. [15]	China	OCT	52	52	52.4	25/27	6.1 years	250/-	SFCT 1500 μm nasal and temporal from the center of the fovea	CT, RT, CCT, IOP< ACD, IT, CBT, VAL, EAL	No Patients with DM were excluded from the study
Coppolino G et al. [16]	Italy	OCTA Optovue	20	35	63.7 ± 11.4	69.2%/ 31.8%	25 months	mean UF rate for an hour never exceeded 0.6 mL/Kg/hour	CCT	SCP VD, DCP VD of whole image, foveal and parafoveal zone, and FAZ for both OCT-A 3 × 3 mm and 6 × 6 mm scans, CRT, central CT	No
Coppolino G et al. [17]	Italy	OCT-A Optovue	15	23	64.3 ± 9.2	66.2%/ 33.8%	27 (16–48) months	300/500	CCT	SCP VD and DCP VD of whole image, foveal and parafoveal zone and FAZ for both OCT-A 3 mm × 3 mm and 6 mm × 6 mm scans CVI, FAZ SCP, central CT	No
Zhang et al. [18]	China	OCT-A Optovue	77	77	53.2 ± 6.8	40/37	4.6 ± 5.1 years	250–300/500	SFCT	SFCT, RT, VD of SCP, DCP, outer retina, choriocapillaris, IOP	Yes
Shin YU et al. [19]	Korea	OCT-A Topcon	29	29	55.6 ± 9.9	12/17	69.3 ± 47.8 months	250/500	nine subfields of the ETDRS grid were obtained, automatically calculated using a macular 3D scan	CT, RT, IOP, VD of SCP, VD of DCP, VD of choriocapillaris	Yes
Hwang et al. [20]	Korea	SD-OCT Heidelberg EDI-mode	15	26	55.93 ± 11.71	6/9	19.19 ± 9.02 years	-/-	SFCT	BCVA, IOP, Central RT, SFCT	No only patients with DM were included in the study
Shoshtari et al. [21]	Iran	OCT Optovue	67	134	57.3 ± 15	40/27	-	250–300/-	SFCT at 500 and 1000 μm intervals temporal and nasal from the fovea	BCVA, IOP refraction, macular thickness, CT	No
Ishibazawa et al. [22]	Japan	SD-OCT Nidek	41 20 DM 21NDM	77	DM: 67.2 ± 7.1 NDM: 66.7 ± 8.7	20/21	DM: 3.9–5.0 years NDM: 5.5–5.3 years	250–300/-	SFCT	IOP, CMT, SFCT	Yes
Chang et al. [23]	Korea	Spectralis OCT Heidelberg	31	54	60.1 ± 7.8	16/15	59.1 ± 29.1 months	250/-	SFCT and 1500 μm temporal to the foveal center and outside the macula in the superior, inferior, and nasal areas 3500 μm from the optic disk margin	VA, IOP, CCT, AL, peripapillary RNFL thickness, CT	Yes
Kang et al. [24]	Korea	EDI OCT Heidelberg	18	-	54.3 ± 9.5	7/11	-	-/-	SFCT	SFCT	Yes

Abbreviations: ACD, anterior chamber depth; AL, axial length; BCVA, best corrected visual acuity; CBT, ciliary body thickness; CCT, central corneal thickness; CFT, central foveal thickness; CMT, central macular thickness; CT, choroidal thickness; CVI, choroidal vascularity index; DCP, deep capillary plexus; DM, diabetic mellitus; EAL, axial length of eye; ECD, endothelial cell density; ECS, the average endothelial cell size; ECSCV, endothelial cell size variation coefficient; EDI, Enhanced Depth Imaging; FAZ, foveal avascular zone; ICA, iridocorneal angle; IOP, intraocular pressure; IT, iris thickness; LA, luminal area; LT, lens thickness; MV, macular volume; NDM, non-diabetes mellitus; OCT-A, optical coherence tomography angiography; OPA, ocular pulse amplitude; RAC, retinal arteriolar caliber; RNFL, retinal nerve fiber layer; RT, retinal thickness; RVC, retinal venular caliber; SA, stromal area; SCP, superficial capillary plexus; SD-OCT, spectral domain optical coherence tomography; SFCT, subfoveal choroidal thickness; SS-OCT, swept-source optical coherence tomography; TBUT, tear break up time; TCA, total choroidal area; VA, visual acuity; VAL, axial length of vitreous; VD, vessel density.

**Table 3:** Changes in systemic parameters for the entire group of the included studies.

Studies	Ultrafiltration Volume (mL)	Body Weight (kg)		Systolic Blood Pressure (mmHg)		Diastolic Blood Pressure (mmHg)		Mean Arterial Pressure (mmHg)		Plasma Colloid Osmotic Pressure		Serum Osmolarity (mOs m/L)		Ocular Perfusion Pressure	
Jung et al. [4]	-	Before: 63.17 ± 9.75 After: 61.01 ± 9.81 *p* < 0.001		Before: 158.68 ± 24.24 After: 142.16 ± 22.64 *p* = 0.018		Before: 78.68 ± 14.26 After: 78.26 ± 14.85 *p* = 0.859		-		Before: 25.12 ± 1.63 After: 29.42 ± 2.6 *p* < 0.001		Before: 312.74 ± 11.87 After: 292.74 ± 7.8 *p* < 0.001		decreased *p* = 0.052	
Kal et al. [5]	3136 ± 578	-		Before: 131.6 ± 13.1 After: 119.0 ± 10.7 *p* < 0.001		Before: 82.6 ± 11.1 After: 70.8 ± 9.3 *p* < 0.001		Before: 98.6 ± 10.6 After: 87.2 ± 9.1 *p* < 0.001		-		-		Before: 48.9 ± 8.5 After: 41.4 ± 7.3 *p* < 0.001	
Çelikay et al. [6]	2647.22 ± 731.43	Before: 65.77 ± 10.5 After: 63.65 ± 10.41 *p* < 0.001		Before: 126.12 ± 19.4 After: 104.49 ± 22.61 *p* < 0.001		Before: 83.24 ± 23.26 After: 69.24 ± 11.6 *p* < 0.001		-		-		-		-	
Mayali et al. [7]	1412.4 ± 748.3	Before: 63.59 ± 10.96 After: 62.34 ± 11.15 *p* < 0.001		Before: 115.45 ± 17.38 After: 108.64 ± 17.81 *p* = 0.04		Before: 68.18 ± 7.95 After: 67.73 ± 7.52 *p* = 0.73		-		-		-		-	
Ulaş et al. [8]	3042.86	-		-		-		Before: 80.33 After: 77.9 *p* = 0.22		Before: 23.66 After: 28.76 *p* < 0.001		-		-	
Yang et al. [9]	-	Δ = 2.8 ± 1.3		Before: 139.3 ± 24.7 After: 131.6 ± 34.2 *p*-not rated		Before: 82.4 ± 15.7 After: 82.8 ± 14.7 *p*-not rated		-		-		-		-	
Chen et al. [10]	-	-		Before: 147.14 ± 22.43 After: 136.09 ± 24.37 *p* = 0.001		Before: 83.75 ± 16.03 After: 76.48 ± 13.47 *p* = 0.006		-		-		-		-	
Nakano et al. [11] *	-	DM	NDM	DM	NDM	DM	NDM	DM	NDM	DM	NDM	DM	NDM	DM	NDM
		Before: 70.1 ± 13.9 After: 66.5 ± 13.5 *p* < 0.01 Δ = 3.63 ± 1.59	Before: 60 ± 11.4 After: 57.8 ± 10.5 *p* < 0.01 Δ = 2.78 ± 2.2	Before: 155.6 ± 16 After: 137.1 ± 16.8 *p* = 0.04	Before: 154 ± 21.3 After: 137.3 ± 12.6 *p* > 0.05	Before: 76.5 ± 15.4 After: 72.6 ± 12.5 *p* > 0.05	Before: 82.8 ± 8.7 After: 74.1 ± 6.2 *p* < 0.05	Before: 102.9 ± 12.9 After: 94.1 ± 12.4 *p* = 0.004	Before: 106.8 ± 13.9 After: 94.5 ± 7.3 *p* = 0.003	Before: 19.9 ± 3 After: 21.4 ± 2.8 *p* = 0.03	Before: 21.3 ± 3.9 After: 22 ± 3.3 *p* > 0.05	Before: 307.4 ± 7.5 After: 299.4 ± 7.7 *p* = 0.024	Before: 314.6 ± 7.6 After: 302.3 ± 5.9 *p* = 0.006	Before: 60.3 ± 11.5 After: 59.4 ± 7.8 *p* > 0.05	Before: 61.2 ± 7.9 After: 51.7 ± 4.8 *p* = 0.001
Shin et al. [12]	3000	Before: 62.6 ± 10.5 After: 60.0 ± 10.5 *p* < 0.05 Δ = 2.6 ± 0.8		Before: 153.4 ± 30.8 After: 142.2 ± 28.1 *p* < 0.05 Δ = 11.3 ± 20.3		Before: 76.5 ± 11.4 After: 79.3 ± 10.7 *p* = 0.267 Δ = 2.8 ± 13.9		Before: 102.1 ± 11.7 After: 100.2 ± 14 *p* = 0.424 Δ = 1.9 ± 13.3		-		-		-	
Elbay et al. [13]	2071.2	Before: 64.53 ± 12.71 After: 62.41 ± 12.25 *p* < 0.001		Before: 140 Second hour:120 *p* < 0.01 After: 120 *p* < 0.001		Before: 80 Second hour: 80 *p* = 0.701 After: 70 *p* = 0.054		-		-		-		-	
Sun et al. [14]	-	Before: 66 ± 11.98 After: 63.15 ± 9.42 *p* < 0.001 Δ = 3.12 ± 1.07		Before: 140.54 ± 24.93 After: 118.49 ± 21.11 *p* < 0.001 Δ = 20.13 ± 16.06		Before: 80.84 ± 14.67 After: 67.41 ± 12.63 *p* < 0.001 Δ = 2.57 ± 11.87		-- -		-		-		--	
Wang et al. [15]	2318	Δ = 2.1		Before: 135.19 ± 17.28 Second hour: 123.28 ± 18.56 *p* <0.05 After: 133.03 ± 18.89 *p* > 0.05		Before: 76.98 ± 14.02 Second hour: 74.26 ± 16.9 After: 77.84 ± 15.07 *p* > 0.05		-		Before: 279.37 ± 6.16 After: 270.54 ± 7.32 *p* < 0.05				Before: 53.75 ± 7.8 Second hour: 50.67 ± 9.89 *p* < 0.01 After: 55.23 ± 9.73 *p* > 0.05	
Coppolino G et al. [17]	-	Before: 77.12 ± 12.32 After: 75.14 ± 11.55 *p* = 0.05		-		-		-		-		-		-	
Zhang et al. [18]	2500 ± 1400	-		Before: 123.7 ± 19.7 After: 116.9 ± 24.6 *p* < 0.05		Before: 73.4 ± 12.8 After: 71.9 ± 16.2 *p* > 0.05		-		-		-		Before: 51.2 ± 8.9 After: 48.8 ± 10.1 *p* < 0.05	
Shin YU et al. [19]	2900 ± 800	Before: 62.4 ± 11.4 After: 59.7 ± 11.4 *p* < 0.001		Before: 152.8 ± 32.5 After: 142.7 ± 29.5 *p* = 0.014		Before: 77.5 ± 11.9 After: 79.8 ± 11.7 *p* = 0.369		Before: 102.6 ± 12.7 After: 100.8 ± 15.3 *p* = 0.476		-		-		-	
Hwang et al. [20]	-	Before: 63.43 ± 15.51 After: 61.57 ± 11.97 *p* = 0.059		Before: 151.12 ± 19.7 After: 144.16 ± 0.41 *p* = 0.194		Before: 80.04 ± 15.03 After: 74.72 ± 11.06 *p* = 0.146		Before: 103.73 ± 16 After: 97.87 ± 8.94 *p* = 0.143		-		-		-	
Ishibazawa et al. [22] ^†^	DM: 2770 ± 630; NDM: 2140 ± 950	DM	NDM	DM	NDM	DM	NDM	DM	NDM	-		-		DM	NDM
		Before: 58.1 ± 9.4 After: 55.5 ± 9.3 *p* < 0.01	Before: 57.7 ± 13 After: 55.8 ± 8.7 *p* < 0.01	Before: 168.5 ± 23.4 After: 151.9 ± 19.8 *p* < 0.01	Before: 155.4 ± 26 After: 143.5 ± 28.8 *p* < 0.05	Before: 77.7 ± 12.1 After: 74.6 ± 9.2 *p* > 0.05	Before: 79.0 ± 16.3 After: 73.2 ± 17 *p* < 0.05	Before: 113.7 ± 22.2 After: 106.3± 18.7 *p* < 0.05	Before: 112.1± 19.4 After: 101.9± 21.9 *p* < 0.05					Before: 62.8 ± 14.5 After: 56.7 ± 13.5 *p* < 0.05	Before: 60.4 ± 12.1 After: 54.0 ± 14 *p* < 0.05
Chang et al. [23]	>2000	Before: 65.7 ± 13 After: 63.4 ± 12.6 *p* < 0.001		Before: 144.1 ± 22.5 After: 129.6 ± 21.1 *p* < 0.001		Before: 78.1 ± 13.9 After: 74.5 ± 16.6 *p* = 0.184		Before: 100.1 ± 14.7 After: 92.8 ± 15.6 *p* = 0.008		-		Before: 317.5 ± 12.02 After: 94.4 ± 11.7 *p* < 0.001		Before: 56.6 ± 10.2 After: 52.9 ± 10.3 *p* = 0.041	
Kang et al. [24]		Before: 61.8 ± 14.1 After: 59.7 ± 13.7		Before: 139 ± 14.6 After: 139.4 ± 21.1		Before: 80.2 ± 10.6 After: 82.8 ± 14.1		-		-		-		-	

Abbreviations: DM, patients with diabetes mellitus; NDM, patients without diabetes mellitus. * Body weight change was similar in the DM group and the NDM group (3.63 ± 1.59 kg; vs. 2.78 ± 2.2 kg; *p* = 0.28); † Body fluid removal was larger in the DM group than in the NDM group (2.77 ± 0.63 L; vs. 2.14 ± 0.95 L; *p* = 0.013).

**Table 4:** Correlations between changes in selected systemic parameters and changes in choroidal thickness.

Studies	Ultrafiltration Volume	Body Weight Change	Systolic Blood Pressure Change	Diastolic Blood Pressure Change	Mean Arterial PRESSURE change	Plasma Colloid Osmotic Pressure Change	Serum Osmolarity Change	Ocular Perfusion Pressure Change
Jung et al. [4]	C	A (negative)	A (negative) * only SBP in multivariate analysis	B	C	A (positive)	A (negative)	C
Kal et al. [5]	C	C	C	C	C	C	C	C
Çelikay et al. [6]	B	B	B	A (positive)	C	B	B	C
Mayali et al. [7]	C	C	C	C	C	C	C	C
Ulaş et al. [8]	B	C	B	B	B	B	B	C
Yang et al. [9]	C	A (positive)	B	B	C	C	C	C
Chen et al. [10]	C	C	C	C	C	C	C	C
Nakano et al. [11]	C	C	C	C	C	C	C	C
Shin et al. [12]	B	B	B	B	B	C	C	C
Elbay et al. [13]	C	B	B	B	C	C	C	C
Sun et al. [14]	B	A (positive)	B	B	C	C	C	C
Wang et al. [15]	A (positive)	C	C	C	C	C	C	C
Coppolino G et al. [16]	C	C	C	C	C	C	C	C
Coppolino G et al. [17]	C	C	C	C	C	C	C	C
Zhang et al. [18]	C	C	B	B	C	C	C	C
Shin YU et al. [19]	A (negative) for the entire group and NDM	B	B	B	B	C	C	C
Hwang et al. [20]	C	C	C	C	C	C	C	C
Shoshtari et al. [21]	C	C	C	C	C	C	C	C
Ishibazawa et al. [22]	A (negative) only for NDM	B	B For DM and NDM	C	C	C	C	B
Chang et al. [23]	C	A (positive)	A (positive)	C	C	C	A (positive)	C
Kang et al. [24]	C	B	B	B	C	C	C	C

A: statistically significant relationship; B: no dependency has been demonstrated; C: not rated. Abbreviations: DM, diabetes mellitus; NDM, non-diabetes mellitus.

## Data Availability

Not applicable.
